# An Efficient Correction Algorithm for Eliminating Image Misalignment Effects on Co-Phasing Measurement Accuracy for Segmented Active Optics Systems

**DOI:** 10.1371/journal.pone.0148872

**Published:** 2016-03-02

**Authors:** Dan Yue, Shuyan Xu, Haitao Nie, Zongyang Wang

**Affiliations:** 1 Changchun Institute of Optics, Fine Mechanics and Physics, Chinese Academy of Sciences, Changchun, China; 2 University of Chinese Academy of Sciences, Beijing, China; Ningbo University, CHINA

## Abstract

The misalignment between recorded in-focus and out-of-focus images using the Phase Diversity (PD) algorithm leads to a dramatic decline in wavefront detection accuracy and image recovery quality for segmented active optics systems. This paper demonstrates the theoretical relationship between the image misalignment and tip-tilt terms in Zernike polynomials of the wavefront phase for the first time, and an efficient two-step alignment correction algorithm is proposed to eliminate these misalignment effects. This algorithm processes a spatial 2-D cross-correlation of the misaligned images, revising the offset to 1 or 2 pixels and narrowing the search range for alignment. Then, it eliminates the need for subpixel fine alignment to achieve adaptive correction by adding additional tip-tilt terms to the Optical Transfer Function (OTF) of the out-of-focus channel. The experimental results demonstrate the feasibility and validity of the proposed correction algorithm to improve the measurement accuracy during the co-phasing of segmented mirrors. With this alignment correction, the reconstructed wavefront is more accurate, and the recovered image is of higher quality.

## Introduction

Segmented active optics (SAO) systems can meet the demands of next-generation space telescopes being lighter, larger and foldable [1]. A segmented primary mirror stitches serial arrays of sub-mirrors together to reach the optimal capabilities of a monolithic mirror. This type of space telescope can be folded for launch and deploy autonomously after reaching orbit [2]. However, high-quality images equivalent to those of a monolithic mirror can only be achieved if co-phasing of the segmented mirrors occurs. Co-phasing of segmented mirrors removes the relative piston aberrations between segments and the tip-tilt aberrations of each segment, making it the one of the core technologies for the practical application of segmented telescopes.

Many methods have been proposed for the co-phasing segmented mirrors to obtain nearly diffraction-limited performance from the total aperture, such as the Mach-Zehnder Interferometer Sensing [3–4], modified Shack-Hartmann Wavefront Sensing (WFS) [5–6], the Curvature Sensing [7–8], the Pyramid Sensing [9–10], the Zernike Phase Contrast Sensing [11] and Phase Diversity WFS (PD WFS) [12–15]. PD WFS stands out in the development of technologies for the co-phasing of SAO systems because traditional wavefront reconstruction using methods such as Shack-Hartmann WFS tend to break down at the mirror segment edges. Additionally, PD WFS does not require new instrumentation to be added to the already complex optical system and is sensitive to both relative piston and tip-tilt aberrations for continuous and discontinuous input of distorted wavefronts.

The basic principle of the PD algorithm is the simultaneous acquisition of a pair of short-exposure images with a known out-of-focus distance to construct an iterative optimization model based on the maximum likelihood estimation theory. Then, the distorted wavefront is reconstructed, and the recovery of an unknown object in the field can be acquired according to the intensity distribution of the gained images. PD WFS requires not only the synchronous acquisition of images but also rigorous alignment of each image. Currently, many refinement approaches of PD technique have been proposed, such as modifying the optimization algorithm to improve the accuracy and noise tolerance [16] or establishing a linear relationship between the detected images and unknown aberrations to improve the speed of the algorithm [17–18]. All these methods are based on utilizing at least two images, which are ideally without misalignment. When there are horizontal and/or vertical position offsets between a pair of in-focus-defocused images, the accuracy of wavefront sensing and the quality of the recovered image will suffer a very serious decline. The strict theoretical demonstration presented in this paper will prove that this image misalignment error precisely corresponds to the tip-tilt aberrations of the wavefront phase. The co-phasing of segmented mirrors eliminates the piston and tip-tilt errors; thus, the strict alignment of the image pair is a compulsory step during the wavefront detection and the recovery of unknown objects based on PD algorithm for the SAO systems.

This paper analyzes the reasons for the appearance of image misalignment errors and gives the strict theoretical proof of the corresponding relationship between these misalignment errors and tip-tilt aberrations in the Zernike polynomials of the wavefront phase. To eliminate the undesirable effects of image misalignment on the wavefront detection accuracy and the object image recovery quality, an efficient two-step alignment correction algorithm is proposed based on the characteristics of PD WFS for SAO systems. This algorithm utilizes a spatial 2-D cross-correlation [19–21] of image pairs to process coarse alignment corrections and restricts the offset to 1 or 2 pixels, which narrows the search range for correction in the next step. Then, adaptive correction is realized without the need for subpixel fine alignment by adding additional tip-tilt terms to the Optical Transfer Function (OTF) of the out-of-focus channel as alignment parameters. A comparison of the results of reconstructed wavefronts and recovered objects with and without alignment correction demonstrates the effectiveness and feasibility of the proposed algorithm and reveals that strict image alignment is indispensable in co-phasing of segmented mirrors to obtain nearly diffraction-limited performance.

## Materials and Methods

### Theory demonstration

The basic theory of the PD algorithm demands acquiring multi-channel images of the same target simultaneously, including images recorded in the in-focus plane and in the out-of-focus plane. The adding channel contains only an out-of-focus aberration introduced by the known out-of-focus amount without any other aberrations. There should not be any position offsets between the recorded images ideally. However, misalignment may occur when fixing the camera or the optical platform in the optical setup, leading to relative offsets in the image pair. Meanwhile, the CCD camera target surface is often larger than the object image; thus, the region of interest (ROI) of the in-focus and out-of-focus images must be intercepted respectively to reduce the amount of the subsequent work, which can also bring about misalignment in the images.

Without loss of generality, single frame images from two-channel are considered here. Consider the image *i*_1_ recorded in the focus plane as a reference; then, the image ${\hat{i}}_{2}$ collected in the out-of-focus plane has relative offsets, which are Δ*u* and Δ*v* in the horizontal and vertical directions, respectively, given by Eq (1):

$${\hat{i}}_{2}=i_{2}(u+\Delta u,v+\Delta v)$$ (1)

where *i*_2_ is the ideal out-of-focus image without any misalignment errors. According to the Fourier transform properties, the formula above can be rewritten as Eq (2) in the frequency domain:

$${\hat{I}}_{2}(f_{u},f_{v})=I_{2}(f_{u},f_{v})e^{j2\pi (f_{u}\Delta u+f_{v}\Delta v)}$$ (2)

By taking the frequency spectrum of the out-of-focus image with misalignment into the PD cost function, we obtain Eq (3):

$$L\left[I_{k};o,\left\{e_{j},T_{xj},T_{yj}\right\}\right]=\sum_{f_{u},f_{v}\in \chi }\left[{\left|I_{1}\right|}^{2}+{\left|{\hat{I}}_{2}\right|}^{2}-\frac{{\left|I_{1}S_{1}{}^{*}+{\hat{I}}_{2}S_{2}{}^{*}\right|}^{2}}{{\left|S_{1}\right|}^{2}+{\left|S_{2}\right|}^{2}+\gamma }\right]$$ (3)

where *S*_1_ and *S*_2_ are OTFs of the in-focus and out-of-focus channels, respectively. The misalignment error of images only affects the numerator of the third term, expanding it to Eq (4):

$${\left|I_{1}S_{1}{}^{*}+{\hat{I}}_{2}S_{2}{}^{*}\right|}^{2}={\left|I_{1}S_{1}{}^{*}+I_{2}S_{2}{}^{*}e^{i2\pi (f_{u}\Delta u+f_{v}\Delta v)}\right|}^{2}={\left|I_{1}S_{1}{}^{*}+I_{2}{\hat{S}}_{2}{}^{*}\right|}^{2}$$ (4)

The purpose of the derivation above is to transfer the misalignment of images to the wavefront phase, thus allowing for a deeper analysis into the OTF of the out-of-focus channel. Eq (5) is obtained using the Fourier phase shift theorem:

$${\hat{S}}_{2}=S_{2}e^{-i2\pi (f_{u}\Delta u+f_{v}\Delta v)}=ℑ\left\{s_{2}(u,v)\right\}e^{-i2\pi (f_{u}\Delta u+f_{v}\Delta v)}=ℑ\left\{s_{2}(u-\Delta u,v-\Delta v)\right\}$$ (5)

Utilizing the relationships between the point spread function (PSF), the impulse response function and the generalized pupil function in incoherent imaging systems, Eqs (6) and (7) can be obtained as

$$s_{2}(u-\Delta u,v-\Delta v)={\left|h_{2}(u-\Delta u,v-\Delta v)\right|}^{2}={\left|{ℑ}^{-1}\left\{{Ρ}_{2}(f_{u},f_{v})e^{-i2\pi (f_{u}\Delta u+f_{v}\Delta v)}\right\}\right|}^{2}$$ (6)

$${\hat{\text{P}}}_{2}={\text{P}}_{2}(f_{u},f_{v})e^{-i2\pi (f_{u}\Delta u+f_{v}\Delta v)}=P_{2}(f_{u},f_{v})e^{i\left[\phi (\rho ,\theta )+\phi_{d}(\rho ,\theta )-2\pi (f_{u}\Delta u+f_{v}\Delta v)\right]}$$ (7)

where *s*_2_ and *h*_2_ are the PSF and impulse response function of the out-of-focus channel, respectively, ${\hat{\text{P}}}_{2}$ and P_2_ are the generalized pupil function with and without misalignment errors, respectively, and *ϕ*(*ρ*,*θ*) and *ϕ*_*d*_(*ρ*,*θ*) denote the co-phase aberrations and constant out-of-focus aberration, respectively. According to the transformation relationship between Cartesian and polar coordinates, a significant conclusion can be drawn here: the image misalignment errors precisely correspond to the tip-tilt terms in Zernike polynomials of the wavefront phase. Thus, through the derivation of the above formulas, image misalignment errors have been mapped to the wavefront aberration of the generalized pupil function, which is the sound theoretical foundation for the proposed correction algorithm.

The image misalignment errors do not affect image quality, namely they have no influence on the 4th- or higher-order Zernike aberrations. However, in the co-phasing of SAO systems, the main aberrations to be removed are the relative piston aberrations between segments and the tip-tilt aberrations of each segment. Thus, for the wavefront detection and image restoration of segmented telescopes based on the PD algorithm, the image pair between the in-focus and out-of-focus planes must be strictly aligned.

### Alignment correction algorithm

The images collected in the in-focus and out-of-focus planes require alignment correction according to the theoretical analysis above. The image in the in-focus plane is typically set as a reference, and the out-of-focus image is aligned to the referenced one. However, the introduced out-of-focus aberrations and the unavoidable noise lead to inefficiency in conventional alignment methods. Aimed at the specific features of SAO systems based on the PD algorithm and the theoretical relationship between misalignment and tip-tilt errors, a two-step alignment correction method is proposed in this paper. First, a spatial 2-D cross-correlation of the in-focus and out-of-focus images is computed to locate a coarse alignment position to narrow the search range. Then, the remaining offsets in the vertical and horizontal directions after coarse alignment are used as search parameters to realize adaptive correction based on the optimization process of solving the PD cost function without subpixel fine alignment.

#### Coarse alignment correction

Many conventional alignment correction approaches cannot yield accurate results such as phase correlation [22] and cross-correlation in the frequency domain [23] due to the introduced out-of-focus aberrations and the presence of a cut-off frequency in the OTFs of the segmented optical system that results in 0 frequency spectrum points of degraded images. This paper processes a spatial 2-D cross-correlation directly to the pixel matrices of images in the in-focus and out-of-focus planes and then locates the position of its maxima. There is a corresponding relationship between coordinate position and image offset [24–26].

The spatial 2-D cross-correlation of a *M* × *N* matrix X and a *P*×*Q* matrix H is a matrix C of size *M* + *P* − 1 by *N* + *Q* − 1 given by Eq (8):

$$C(k,l)=\sum_{m=0}^{M-1}\sum_{n=0}^{N-1}X(m,n)\bar{H}(m-k,n-l)$$ (8)

where −(*P*−1)≤*k*≤*M*−1, −(*Q*−1)≤*l*≤*N*−1 and the bar over H denotes complex conjugation. Assume that matrix X is the template and (*xpeak*, *ypeak*) is the coordinate position of peak point of *C*(*k*, *l*); then, the coarse number of offset pixels can be gained by Eq (9):

$$\begin{array}{c} row\_shift=xpeak-M\\ column\_shift=ypeak-N \end{array}$$ (9)

With coarse alignment correction, the misalignment between the image pair can be limited to 1 or 2 pixels, which narrows the search range for subsequent adaptive alignment correction and improves the computation efficiency and accuracy.

#### Adaptive alignment correction

After coarse alignment, there exists a 1- or 2-pixel offset between the in-focus and out-of-focus images; this offset still has a serious effect on the wavefront detection accuracy and image restoration quality. According to the theoretical proof, image misalignment errors can be mapped to the wavefront phase of the generalized pupil function corresponding to the tip-tilt terms in the Zernike polynomials. Therefore, here, additional tip-tilt terms are added to the Zernike polynomials of original wavefront phase to match the offset between the image pair without requiring subpixel fine alignment. Adaptive correction can be achieved by the optimization algorithm of solving the PD cost function.

The cost function of segmented optics system based on the PD algorithm is expressed as Eq (10):

$$L=\sum_{f_{u},f_{v}\in \chi }\left[\sum_{k}{\left|I_{k}\right|}^{2}-\frac{\sum_{k}{\left|I_{k}S_{k}{}^{*}\right|}^{2}}{\sum_{k}{\left|S_{k}\right|}^{2}+\gamma }\right]$$ (10)

where *I*_1_ and *I*_2_ represent the frequency spectra of in-focus and out-of-focus images with misalignment errors, respectively. In the practical iteration process, the in-focus image is set as reference by default adding no tip-tilt terms. Then, the modified OTFs with matching terms are given in Eq (11):

$$\begin{array}{c} S_{1}=ℑ\left\{{\left|ℑ\left\{\sum_{n=1}^{N}p_{n}(\varepsilon ,\eta )\text{exp}\left[i\frac{2\pi }{\lambda }\left(E_{n}\;*\;Z_{0}+T_{xn}\;*\;Z_{1}+T_{yn}\;*\;Z_{2}\right)\right]\right\}\right|}^{2}\right\}\\ S_{2}=ℑ\left\{{\left|ℑ\left\{\sum_{n=1}^{N}p_{n}(\varepsilon ,\eta )\text{exp}\left[i\frac{2\pi }{\lambda }\left(\begin{array}{l} E_{n}\;*\;Z_{0}+T_{xn}\;*\;Z_{1}+T_{yn}\;*\;Z_{2}+D_{n}\;*\;Z_{3}\\ +\Delta T_{xn}\;*\;Z_{1}+\Delta T_{yn}*Z_{2} \end{array}\right)\right]\right\}\right|}^{2}\right\} \end{array}$$ (11)

where *n* is the index of sub-aperture, *N* is the total number of sub-mirrors, and *Z*_0_, *Z*_1_, *Z*_2_ and *Z*_3_ correspond to piston, tip, tilt and defocus aberrations in Zernike polynomials, respectively. *E*_*n*_, *T*_*xn*_, *T*_*yn*_ and *D*_*n*_ are the corresponding Zernike polynomial coefficients of the *nth* sub-mirror, respectively. Δ*T*_*xn*_ and Δ*T*_*yn*_ are the additional tip-tilt matching terms. During each iteration of the search process, the OTFs of the in-focus and out-of-focus channels in Eq (11) are used to compute the cost function to perform nonlinear constraints. Thus, the tip-tilt errors introduced by the misalignment between images can be eliminated automatically by the PD optimization algorithm without the subpixel fine alignment, and then, the real aberration coefficients are obtained.

The OTFs used to recover the object image also need the additional matching tip-tilt terms to obtain the correct result. Taking the final searched coefficients of the actual aberrations and the matching tip-tilt terms to Eq (11), the OTFs of the corresponding channels are obtained and the recovery of the object is achieved by Eq (12):

$$O=\frac{\sum_{k=1}^{K}I_{k}S_{k}{}^{*}}{\sum_{k=1}^{K}{\left|S_{k}\right|}^{2}+\gamma }$$ (12)

**Fig 1:**
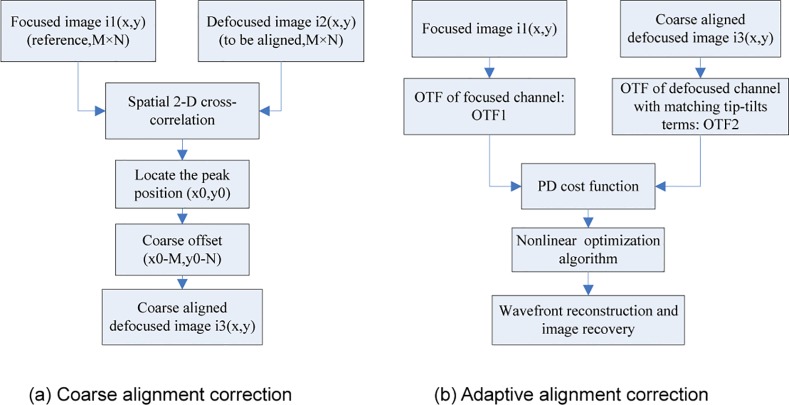
Flowchart of the proposed alignment correction algorithm.

The flowchart for the alignment correction method is shown in Fig 1.

## Results

In this section, the effectiveness and accuracy of the proposed alignment correction algorithm are demonstrated using several numerical simulations.

**Fig 2:**
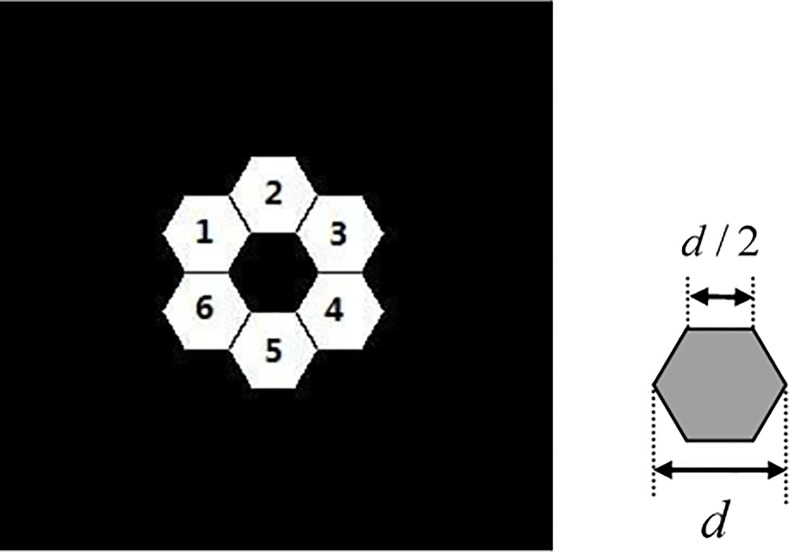
Construction of primary mirror and dimension of segmented sub-aperture.

The parameters of the optical system used in the simulation are as follows. The segmented primary mirror consists of 6 hexagon sub-mirrors; their construction and sequence are shown in Fig 2. The hexagon sub-mirror diameter is *d* and occupies 43×43 pixels in the pupil plane. The diameter of the primary mirror is *D*, and its corresponding pixels are 128×128. To satisfy Nyquist sample theory, the entire pupil plane is set to 256×256 pixels. The *F*^#^ of the optical system is 8, the monochromatic wavelength is 570*nm* and the out-of-focus distance is set to 400*λ*.

**Fig 3:**
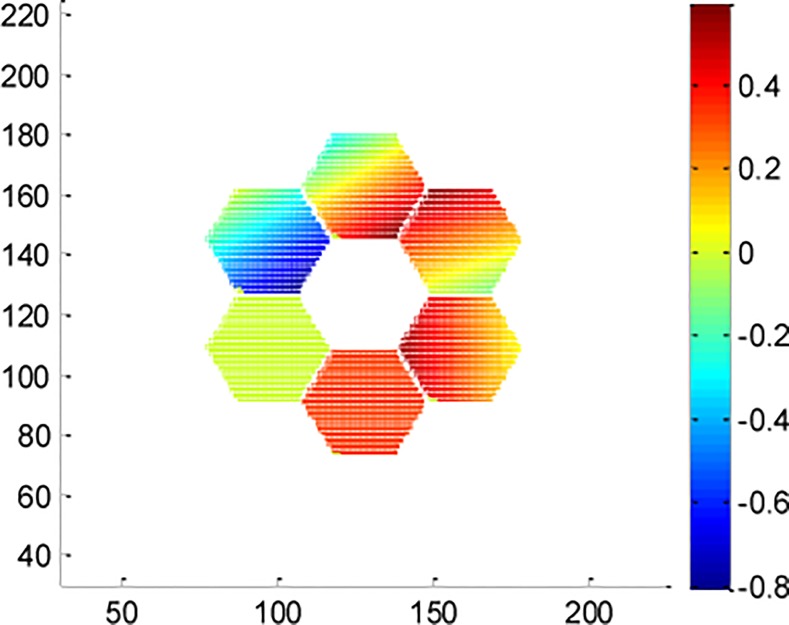
Phase distribution of original distorted wavefront of the resolution test panel experiment.

The 1st sub-mirror is set as the standard mirror; then, a set of random piston and tip-tilt errors listed in Table 1 are applied to all sub-apertures with co-phase errors restricted within ±0.5*λ*. The resulting phase distribution of the distorted wavefront is shown in Fig 3.

**Table 1:** A set of random co-phase errors for simulation.

Index of sub-mirrors	Piston/*λ*	Tip/*λ*	Tilt/*λ*
**1**	0	0	0
**2**	0.33	0	0
**3**	0.28	0	-0.26
**4**	0.25	0.34	-0.13
**5**	0.13	-0.38	0.24
**6**	-0.39	0.35	-0.23

First, consider that the object image only occupies part of CCD target surface; specifically, the object is located in the middle of the target surface and is distinct from the background. Panoramic images will be discussed later.

**Fig 4:**
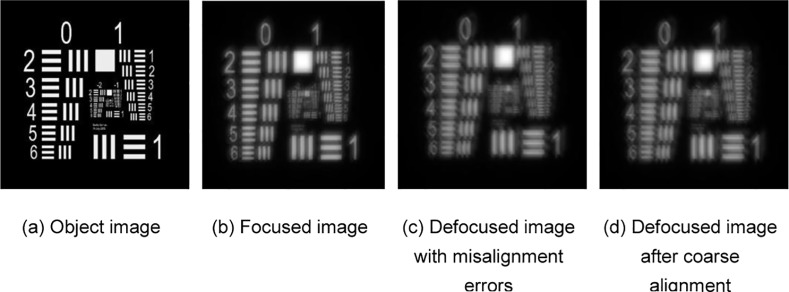
Relevant object images of the resolution test panel experiment.

**Fig 5:**
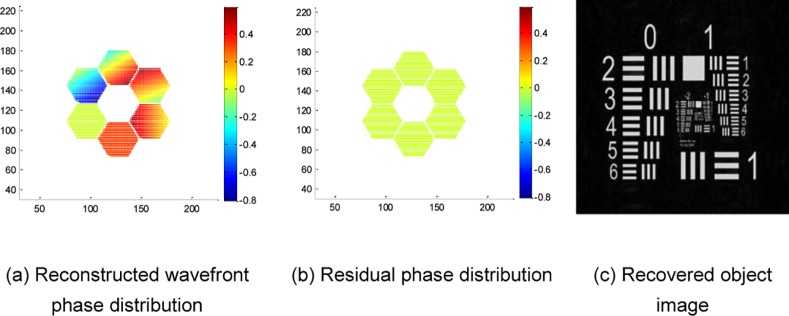
Experimental results of the resolution test panel with alignment correction.

**Fig 6:**
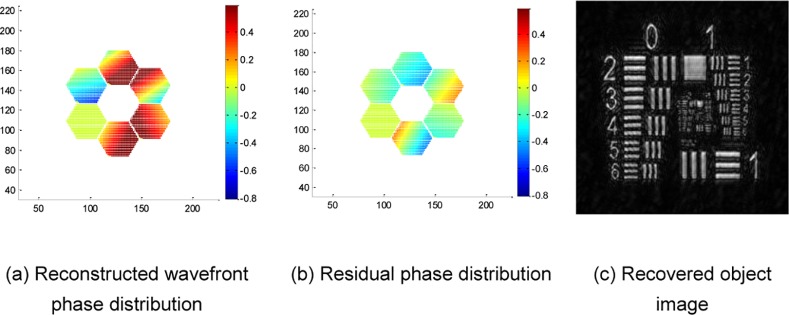
Experimental results of the resolution test panel without alignment correction.

Take the commonly used resolution test panel in laboratory shown in Fig 4(A) as an observed object; then, the in-focus image and out-of-focus image with misalignment errors obtained by the SAO simulation system are given by Fig 4(B) and 4(C), respectively. The coarse aligned out-of-focus image is shown in Fig 4(D), which limits the misalignment error within the range of 1–2 pixels. Then, the adaptive alignment correction is processed by using the in-focus image in Fig 4(B) and coarse aligned out-of-focus image in Fig 4(D) to realize adaptive correction. This paper utilizes L-BFGS as a nonlinear optimization algorithm to solve the PD cost function. The reconstructed wavefront aberration coefficients are listed in Table 2. The reconstructed wavefront phase distribution, residual phase distribution and recovered object with alignment correction are shown in Fig 5(A), 5(B) and 5(C), respectively. To show the contrast effect, the experimental results without alignment correction are given in Fig 6. Fig 6(A) and 6(B) show the reconstructed wavefront phase distribution and residual phase distribution, respectively. Fig 6(C) presents the recovered object without correction. The contrast experimental results show that without alignment correction, the reconstructed wavefront greatly deviates from the actual wavefront; the recovered object image has lower contrast, more blurred edges and the same inclination trend with the misalignment errors.

**Table 2:** Reconstructed wavefront aberration coefficients and residual errors.

Index of sub-mirror	Piston/*λ*			Tip/*λ*			Tilt/*λ*		
	*p*_*j*0_	*p*_*j*_	Δ*p*_*j*_	*tx*_*j*0_	*tx*_*j*_	Δ*tx*_*j*_	*ty*_*j*0_	*ty*_*j*_	Δ*ty*_*j*_
**1**	0	0	0	0	0	0	0	0	0
**2**	0.33	0.3300	0.0225 e-03	0	-0.0000	0.0342 e-03	0	-0.0001	0.1031 e-03
**3**	0.28	0.2799	0.1422 e-03	0	-0.0000	0.0027 e-03	-0.26	-0.2600	0.0368 e-03
**4**	0.25	0.2498	0.2404 e-03	0.34	0.3400	0.0256 e-03	-0.13	-0.1301	0.0646 e-03
**5**	0.13	0.1298	0.2045 e-03	-0.38	-0.3800	0.0187 e-03	0.24	0.2399	0.0581 e-03
**6**	-0.39	-0.3901	0.0917 e-03	0.35	0.3500	0.0393 e-03	-0.23	-0.2301	0.0792 e-03

**Fig 7:**
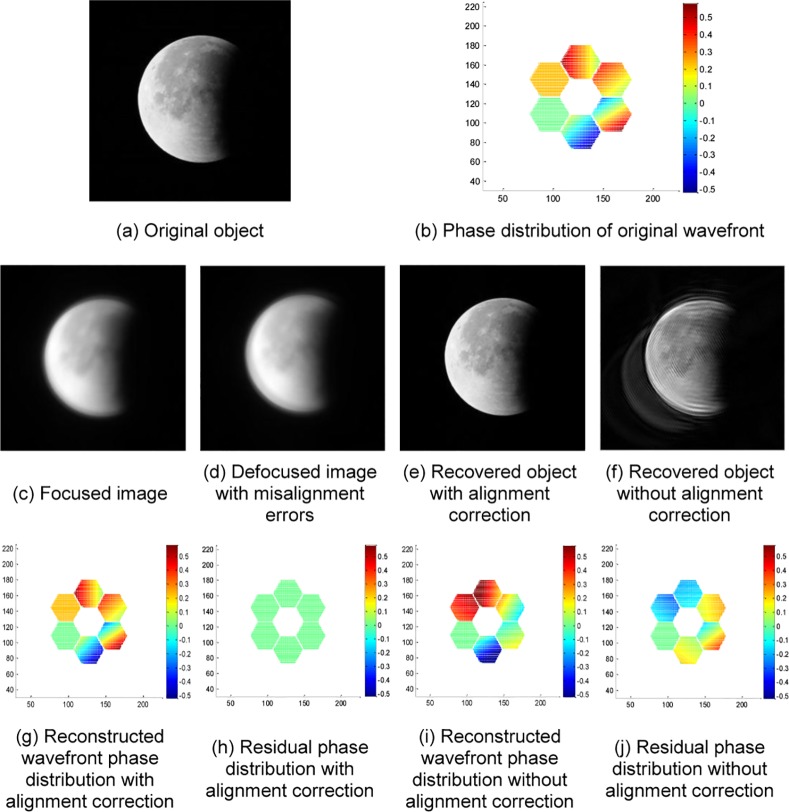
Experimental results of an actual image of the lunar eclipse.

An actual image of lunar eclipse taken by a Maca telescope is used as another observed object for jointly estimating the wavefront and object image under the larger aberrations of the SAO simulation systems. The experimental results are shown in Fig 7.

**Fig 8:**
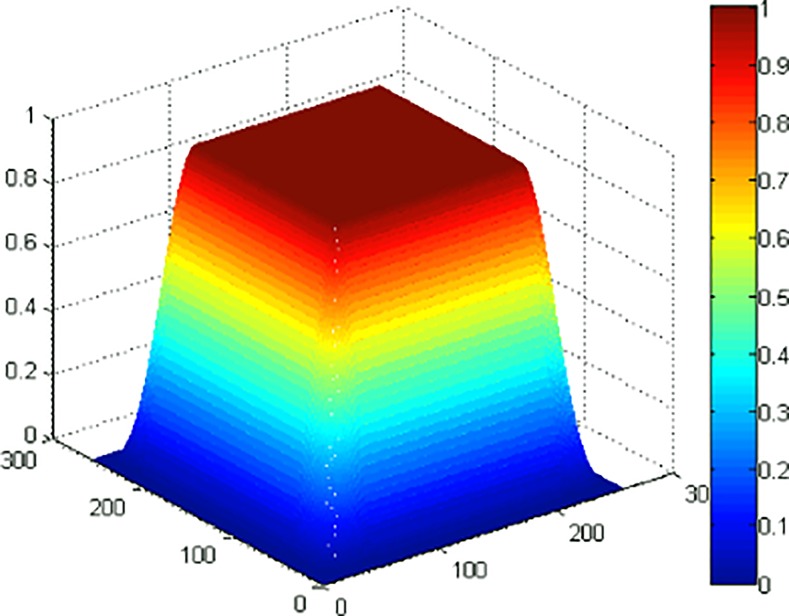
Modified 2-D Hanning window.

**Fig 9:**
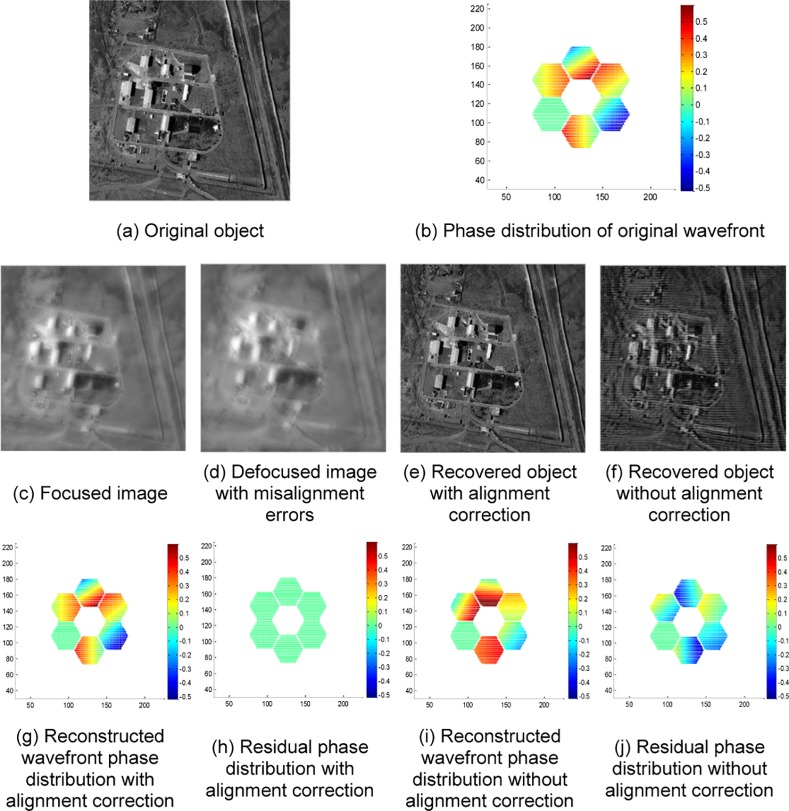
Experimental results of a satellite map of military base.

**Fig 10:**
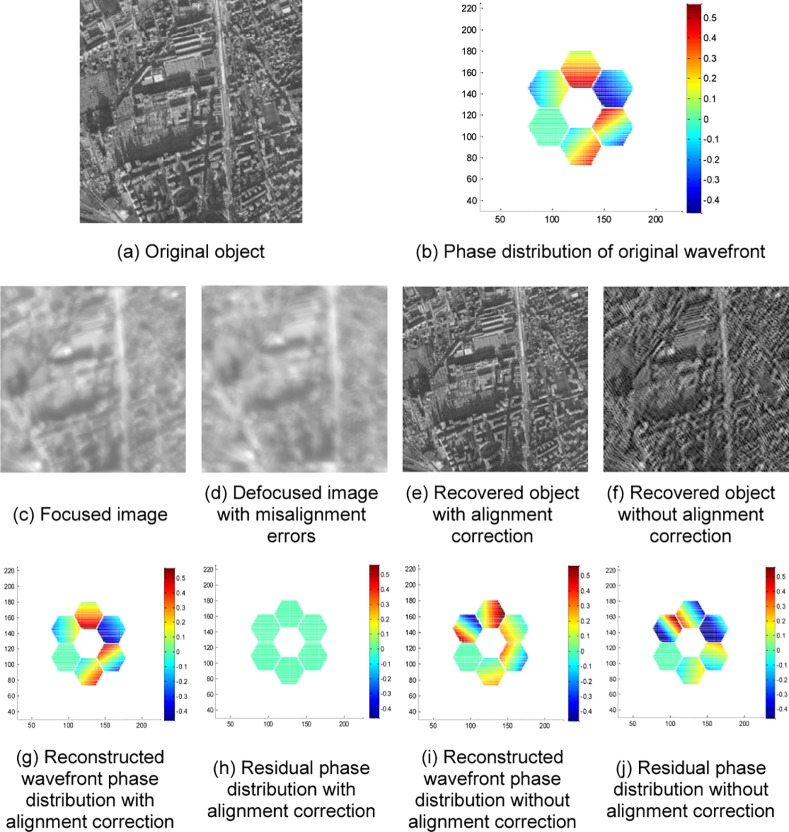
Experimental results of a satellite map of urban scene.

For panoramic images, a window function should be added to images before alignment correction. The goal is to weaken the misalignment image edge, improve the alignment accuracy and suppress the Fourier cycle edge effect. The size and type of the chosen window function depends on the object image and the PSF. A modified 2-D Hanning window, shown in Fig 8, is used in this paper for panoramic images and is added to the observed images. The experimental results of a satellite map of a military base are shown in Fig 9. Another experiment using a satellite map of an urban scene with dense texture is also tested to verify the alignment algorithm. The results are given by Fig 10.

To evaluate the wavefront detection accuracy and the image restoration quality, the following part presents the corresponding evaluation indicators:

1. The root-mean-square-error (*RMSE*) for wavefront detection:
  $$RMSE=\sqrt{\frac{\sum_{i=1}^{M}\sum_{j=1}^{N}{\left(\phi -\phi_{0}\right)}^{2}}{M\times N}}$$ (13)
  where *ϕ*_0_ is the simulated phase distribution needing to be measured and *ϕ* is the reconstructed phase distribution by the PD algorithm. *M* and *N* denote the sampling number. A smaller *RMSE* value indicates a higher wavefront detection accuracy.
2. *PV* value: peak-to-valley value of the phase. A smaller *PV* value means a smaller residual error.
3. Mean-square-error (*MSE*) for image restoration:
  $$MSE=\frac{\sum_{i=1}^{M}\sum_{j=1}^{N}{\left[f(i,j)-\hat{f}(i,j)\right]}^{2}}{M\times N}$$ (14)
  where *f*(*i*, *j*) and $\hat{f}(i,j)$ represent the pixel values at point (*i*, *j*) of the reference image and the image to be evaluated, respectively. *M* and *N* denote the sampling number. A smaller *MSE* value indicates a better image quality.
4. Similarity Measurement (*SM*):
  $$SM=\frac{\sum_{i=1}^{M}\sum_{j=1}^{N}f(i,j)\hat{f}(i,j)}{{\left[\sum_{i=1}^{M}\sum_{j=1}^{N}f^{2}(i,j)\sum_{i=1}^{M}\sum_{j=1}^{N}{\hat{f}}^{2}(i,j)\right]}^{1/2}}$$ (15)
  *SM* utilizes the similarity degree of the gray value of the two images to indirectly assess the image restoration effect. A value close to 1 indicates that the recovered image better approximates the original object, indicating that the image quality is better.

The evaluation results of the wavefront detection accuracy and image restoration quality according to the defined indicators are listed in Table 3. Table 3 shows that with alignment correction, the *RMSE* and *PV* values of the residual phase are considerably smaller than those without correction and are within the acceptable range for SAO systems. For image restoration, the *MSE* value with alignment correction is smaller, indicating that the recovered image is of better quality. The *SM* value is close to 1, implying that the restored image has a higher similarity degree and lower deviation from the ideal image than that without correction.

**Table 3:** Evaluation results of the wavefront detection accuracy and image restoration quality.

Object Indicators		Resolution test panel		Lunar eclipse		Military base		Urban scene	
		With correcti-on	Without correcti-on	With correcti-on	Without correcti-on	With correcti-on	Without correcti-on	With correcti-on	Without correcti-on
**Wave-front Detec-tion**	*RMSE* (*λ*)	6.1837e-05	0.0495	4.5327e-05	0.0554	3.5853e-05	0.0628	2.2918e-05	0.0754
	*PV* (*λ*)	4.6500e-04	0.7416	3.8451e-04	0.7584	3.5682e-04	0.8910	1.9000e-04	1.0352
**Image Resto- ration**	*MSE*	18.2009	35.1929	2.1157	37.3170	59.5289	176.3406	61.3926	144.3692
	*SM*	0.8942	0.6354	0.9859	0.7813	0.7968	0.4215	0.7965	0.3924

## Discussion

For the co-phasing of SAO systems, the main task is to eliminate piston and tip-tilt errors. The PD algorithm is typically used to detect the discontinuous wavefront; however, misalignment of the images recorded in the in-focus and out-of-focus planes will lead to a serious decline of the wavefront detection accuracy and image restoration quality. To solve this problem, this paper rigorously demonstrated the theoretical relationship between image misalignment and the tip-tilt terms in the Zernike polynomials of the wavefront phase and then proposed an efficient two-step alignment correction algorithm. This algorithm processes a spatial 2-D cross-correlation to an image pair with misalignment errors to achieve a coarse alignment correction, which narrows the search range for subsequent correction. Then, additional tip-tilt terms are added to the OTF of the out-of-focus channel as search parameters to realize adaptive correction without the need for subpixel fine alignment. The experimental results of both the object image distinct from the background and panoramic image demonstrate the effectiveness and veracity of the proposed alignment correction algorithm, with the reconstructed wavefront being more accurately determined and the recovered image being closer to the ideal object, resulting in higher quality.
